# Novel Self-Assembly-Induced Gelation for Nanofibrous Collagen/Hydroxyapatite Composite Microspheres

**DOI:** 10.3390/ma10101110

**Published:** 2017-09-21

**Authors:** Jae-Won Choi, Jong-Woo Kim, In-Hwan Jo, Young-Hag Koh, Hyoun-Ee Kim

**Affiliations:** 1 Department of Biomedical Engineering, Korea University, Seoul 02841, Korea; bblackcat@naver.com (J.-W.C.); hiidong98@naver.com (J.-W.K.); jojotan@naver.com (I.-H.J.); 2 Department of Materials Science and Engineering, Seoul National University, Seoul 08826, Korea; kimhe@snu.ac.kr

**Keywords:** biomaterials, porous scaffolds, collagen, hydroxyapatite, *in vitro* bioactivity

## Abstract

This study demonstrates the utility of the newly developed self-assembly-induced gelation technique for the synthesis of porous collagen/hydroxyapatite (HA) composite microspheres with a nanofibrous structure. This new approach can produce microspheres of a uniform size using the droplets that form at the nozzle tip before gelation. These microspheres can have a highly nanofibrous structure due to the immersion of the droplets in a coagulation bath (water/acetone), in which the collagen aggregates in the solution can self-assemble into fibrils due to pH-dependent precipitation. Bioactive HA particles were incorporated into the collagen solutions, in order to enhance the bioactivity of the composite microspheres. The composite microspheres exhibited a well-defined spherical morphology and a uniform size for all levels of HA content (0 wt %, 10 wt %, 15 wt %, and 20 wt %). Collagen nanofibers—several tens of nanometers in size—were uniformly present throughout the microspheres and the HA particles were also well dispersed. The *in vitro* apatite-forming ability, assessed using the simulated body fluid (SBF) solution, increased significantly with the incorporation of HA into the composite microspheres.

## 1. Introduction

Collagen is the major component of the extracellular matrix (ECM) and plays a crucial role in the formation of both hard (e.g., bone and tooth) and soft tissues (e.g., cartilage, skin, muscle, and tendon) [1,2]. Thus, collagen-based biomaterials have been extensively used in tissue regeneration due to their excellent cell signaling properties, good biocompatibility, and desirable biodegradability *in vivo* [3]. In particular, they can be used as templates to induce the precipitation of apatite crystals, which can allow for the production of organic/inorganic composites mimicking the composition and structure of natural bones, thus finding very useful applications for bone regeneration [4,5,6,7,8]. In addition, when shaped into microspheres, they can be used as carriers for the delivery of bioactive molecules, such as drugs, growth factors, and cells, as well as scaffolds for tissue regeneration and injectable fillers [9,10,11,12]. 

Emulsion is one of the most widely used techniques for the synthesis of polymeric microspheres owing to the simplicity of the process [13,14,15]. In this technique, droplets of a polymer solution can be dispersed in a stable manner in an immiscible liquid medium; when followed by gelation, well-defined microspheres are formed. Recently, several techniques have been developed to create porous collagen microspheres [9,16], including modified emulsification [10,17] and thermally induced phase separation [18,19,20]. Porous microspheres can have much larger surface areas and longer paths for mass transport, thus offering significantly enhanced functions when used as scaffolds and drug carriers [9,16,21]. However, only a few attempts have been made to mimic the unique nanostructure of natural ECM and its excellent tissue regeneration ability in vivo.

**Figure 1:**
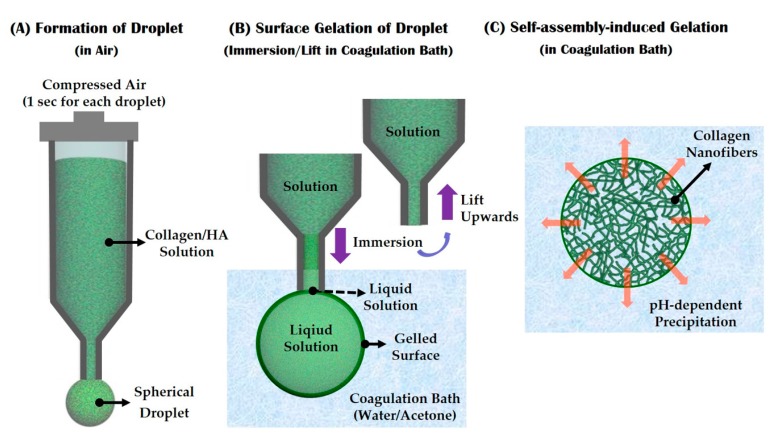
Schematic diagrams showing self-assembly-induced gelation technique for the synthesis of nanofibrous collagen/ hydroxyapatite (HA) composite microspheres: (**A**) The formation of a droplet at the nozzle tip; (**B**) Surface gelation of the droplet after immersion in a coagulation bath, followed by an upwards lift to detach the droplet from the nozzle tip; (**C**) Self-assembly-induced gelation in the coagulation bath to create a nanofibrous structure.

We herein propose a novel and simple way of synthesizing porous collagen-based microspheres, which can have an ECM-mimicking nanofibrous structure, using the self-assembly-induced gelation process. An overview of this approach is shown in Figure 1. A collagen solution is dispensed through a fine nozzle into the air for a very short period of time, in order to form a droplet at the tip (Figure 1A). The droplet is then immersed in a coagulation bath (water/acetone), in which the outer layer of the collagen droplet can be rapidly gelled, while the solution at the end of the nozzle remains liquid (Figure 1B). The droplet can be readily detached from the tip when the nozzle is lifted upwards, after which it can be gelled via pH-dependent precipitation in the coagulation bath [22,23,24,25,26]. This process can effectively induce the self-assembly of collagen aggregates into fibrils, resulting in an entangled network of collagen fibrils and nanopores (Figure 1C). 

To demonstrate the utility of the present approach, nanofibrous collagen/hydroxyapatite (HA) composite microspheres were synthesized, in which bioactive HA phase was added to the collagen in order to enhance bone regeneration ability [9,27,28,29]. Various collagen/HA composite microspheres with different levels of HA content (0 wt %, 10 wt %, 15 wt %, and 20 wt %) were synthesized and their morphology, micro/nano-structure, and chemical composition were evaluated using several analysis tools. The *in vitro* apatite-forming ability of the collagen/HA composite microspheres was also examined to evaluate their potential applications in bone tissue regeneration.

## 2. Materials and Methods

### 2.1. Starting Materials

Unless specified otherwise, all reagents were purchased from Sigma-Aldrich (St. Louis, MO, USA). Collagen powder (Sewon Cellontech Co., Ltd., Seoul, Korea), Type I atelocollagen was used without further purification. Commercially available hydroxyapatite (HA) powder (OssGen Co., Gyeongsan-si, Korea) with a mean size of 0.5 μm was used as the filler.

### 2.2. Collagen/HA Composite Solutions Preparation

To prepare the collagen/HA composite solution, a pure collagen solution with a concentration of 2.5 *w*/*v* was first prepared by dissolving collagen powder in 0.05 M acetic acid at a pH of ~4.2 under magnetic stirring for 24 h. After this, predetermined amounts of HA powder (0 wt %, 5 wt %, 10 wt %, and 20 wt % in relation to the collagen) were added to the prepared collagen solutions and then stirred for 24 h.

### 2.3. Self-Assembly-Induced Gelation

Nanofibrous collagen/HA composite microspheres with different levels of HA content (0 wt %, 10 wt %, 15 wt %, and 20 wt %) were synthesized using self-assembly-induced gelation (Figure 1). To accomplish this, the coagulation batch was filled with a water/acetone (87.5:12.5, *v*/*v*) mixture containing ammonium hydroxide. The final pH of the coagulation medium was adjusted to ~10.2 with HCl.

The prepared collagen/HA composite solution was loaded into a 15 cc syringe and then dispensed through a fine nozzle with a diameter of ~250 μm in air to form a droplet at the tip (Figure 1A). Air pressure of 45 kPa was applied for 1 s using a syringe dispenser (U300; U-jin tech, Co., Ltd., Siheung-si, Korea). After which, the nozzle tip having the droplet was immersed in the coagulation bath and then lifted upwards to detach the droplet from the tip (Figure 1B). The collagen/HA droplet in the coagulation bath was left for 30 min under magnetic stirring, in order to induce gelation via pH-dependent precipitation (Figure 1C).

### 2.4. Cross-Linking and Freeze Drying

The collagen/HA composite microspheres synthesized using different levels of HA content (0 wt %, 10 wt %, 15 wt %, and 20 wt %) were immersed in ethanol used as a dehydration medium containing a 1-ethyl-3-(3-dimethyl aminopropyl) carbodiimide (EDC) (0.05 M) as the chemical cross-linker for 24 h. After immersion in water for 15 min, the collagen/HA composite microspheres were freeze dried for 24 h to completely remove residual solvents (i.e., water and ethanol).

### 2.5. Morphology, Microstructure, and Nanostructure Characterization

The morphology, microstructure, and nanostructure of the nanofibrous collagen/HA composite microspheres synthesized using different levels of HA content (0 wt %, 10 wt %, 15 wt %, and 20 wt %) were analyzed by field emission scanning electron microscopy (FE-SEM; JSM-6701F; JEOL Techniques, Tokyo, Japan). The size distribution of the composite microspheres was approximately evaluated by measuring the diameter of individual microspheres based on the SEM images. Approximately 30 microspheres were measured for each test to obtain an average and standard deviation.

### 2.6. Chemical Composition and Crystalline Phase Analyses

The chemical compositions of the nanofibrous collagen/HA composite microspheres were determined by energy dispersive spectroscopy (EDS) attached to the SEM. Their crystalline phases were characterized by X-ray diffraction (XRD, M18XHF-SRA, MacScience Co., Yokohama, Japan).

### 2.7. TGA Analysis

The final content of the HA particles in the collagen/HA composite microspheres was determined by thermogravimetric analysis (TGA; TA Instruments, New Castle, DE, USA). The composite microspheres were heated up to 700 °C at a heating rate of 10 °C/min in a flowing nitrogen atmosphere. The weight loss of the composite microspheres during the tests was monitored and used to calculate the final HA contents.

### 2.8. In Vitro Apatite-Forming Ability Evaluation

The *in vitro* apatite-forming bioactivity of the nanofibrous the nanofibrous collagen/HA composite microspheres synthesized using different levels of HA (0 wt %, 10 wt %, 15 wt %, and 20 wt %) was characterized using a stimulated body fluid (SBF) solution. The SBF was prepared according to the method reported in the literature [30,31]; its ion concentration is summarized in Table 1. The composite microspheres were immersed in SBF and then placed inside an incubator at a controlled temperature of 37 °C for 3 days. The formation of apatite layers on the composite microspheres was examined by FE-SEM, EDS, and XRD. 

### 2.9. Bioactive Molecule-Loaded Microsphere Synthesis and Evaluation

In order to demonstrate the utility of the present approach for the synthesis of nanofibrous collagen microspheres as carriers for the delivery of bioactive molecules, green fluorescent protein (GFP), produced using *E. coli* [32], was used as the model biomolecule. A green fluorescent protein (GFP) solution with a concentration of 100 μg/mL was directly incorporated into a collagen solution. The final content of the GFP in the collagen solution was 7.0 μg/mL. The morphology of the GFP-loaded collagen microspheres was evaluated by SEM and the distribution of the GFP in the microspheres was characterized by confocal laser scanning microscopy (CLSM), where the green fluorescence can be considered as an indication of the GFP.

In addition, a pre-osteoblast cell line (MC3T3-E1; ATCC, CRL-2593, Rockville, MD, US) was used to demonstrate the potential of the present approach for the synthesis of cell-loaded collagen microspheres. The preincubated cells with a density of 2 × 10^5^ cells/mL were mixed with a collagen solution (3 mL). This cell-loaded solution was directly used to synthesize cell-loaded collagen microspheres. The morphology of the cells incorporated into the as-synthesized microspheres before post-treatments (e.g., crosslinking and freeze-drying) was examined by CLSM. For these CLSM observations, the cells were dyed with Alexa Fluor 546 phalloidin (Eugene, OR, USA) and ProLong Gold antifade reagent with DAPI (Eugene, OR, USA). The stained microspheres were placed on a cover slide, and the cell morphology was observed.

## 3. Results and Discussion

### 3.1. Morphology of Collagen/HA Composite Microspheres

**Figure 2:**
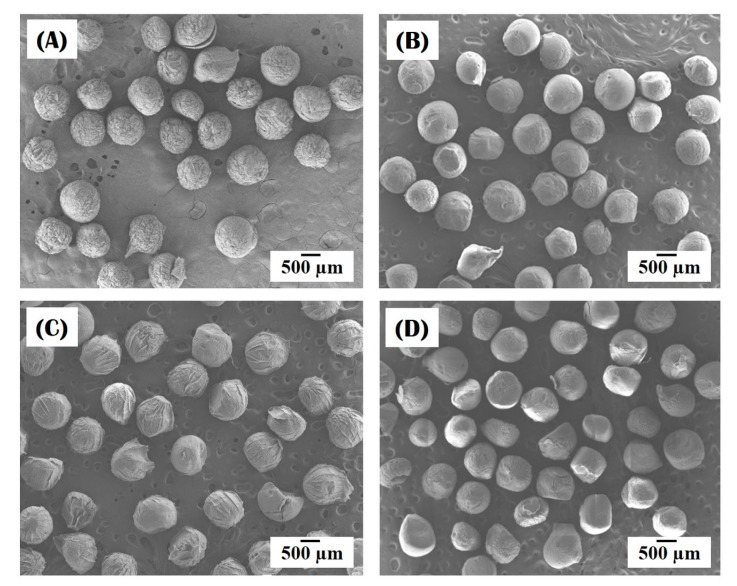
Representative field emission scanning electron microscopy (FE-SEM) images of the morphology of nanofibrous collagen/HA composite microspheres synthesized with different levels of HA: (**A**) 0 wt %; (**B**) 10 wt %; (**C**) 15 wt %; (**D**) 20 wt %.

Nanofibrous collagen/HA composite microspheres were successfully synthesized using a novel self-assembly-induced gelation technique. Figure 2A–D present the morphology of the nanofibrous collagen/HA composite microspheres synthesized using different levels of HA content (0 wt %, 10 wt %, 15 wt %, and 20 wt %) in the collagen/HA solutions. For all levels of HA, the composite microspheres demonstrated a well-defined spherical morphology. 

The diameters of the composite microspheres, measured from the SEM images, are summarized in Table 2. All the composite microspheres had similar diameters with a narrow size distribution: 802 μm ± 40 μm, 798 μm ± 42 μm, 806 μm ± 38 μm, and 809 μm ± 34 μm for HA content of 0 wt %, 10 wt %, 15 wt % and 20 wt %, respectively. This uniformity in size is due to the controlled formation of droplets via the extrusion of the collagen-based solutions through a fine nozzle in air (cf. Figure 1), an important feature of self-assembly-induced gelation that is not shared by conventional emulsion techniques. 

### 3.2. Nanofibrous Structure of Collagen/HA Composite Microspheres

**Figure 3:**
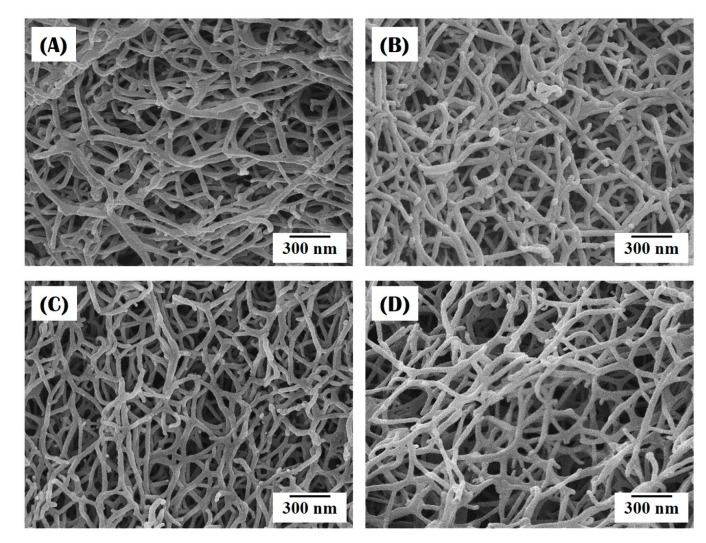
Representative FE-SEM images of the nanofibrous structure of nanofibrous collagen/HA composite microspheres synthesized with different levels of HA: (**A**) 0 wt %; (**B**) 10 wt %; (**C**) 15 wt %; (**D**) 20 wt %.

The nanofibrous structure of the collagen/HA composite microspheres synthesized using different levels of HA content (0 wt %, 10 wt %, 15 wt %, and 20 wt %) was characterized by FE-SEM, as shown in Figure 3A–D. Regardless of the HA level, all the composite microspheres had a highly nanofibrous structure. A number of collagen nanofibers—several tens of nanometers in size—were uniformly formed throughout the microspheres. It should be noted that the creation of the nanofibrous structure, which is one of the most striking features of self-assembly-induced gelation, is attributed to the unique phase separation associated with a pH-dependent precipitation mechanism in the coagulation bath [24,25,26]. More specifically, the collagen aggregates in the collagen solution (pH ~ 4.2) can self-assemble into fibrils when immersed in the coagulation bath (pH ~ 10.2), resulting in an entangled network of collagen fibrils.

### 3.3. Incorporation of HA Particles into Composite Microspheres

**Figure 4:**
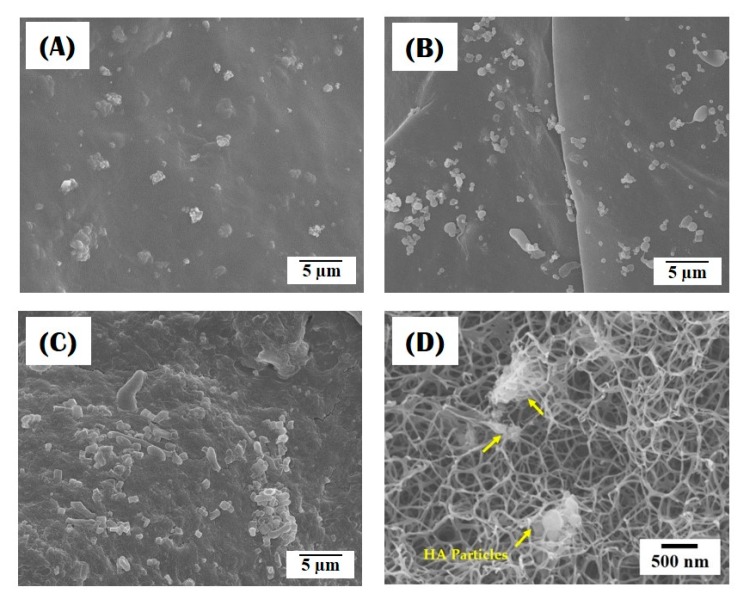
Representative FE-SEM images showing the distribution of HA particles in nanofibrous collagen/HA composite microspheres synthesized with different levels of HA: (**A**) 10 wt %; (**B**) 15 wt %; (**C**,**D**) 20 wt %.

Figure 4A–D present the distribution of HA particles within the nanofibrous collagen/HA composite microspheres synthesized using different levels of HA content (10 wt %, 15 wt %, and 20 wt %). All the composite microspheres revealed that the HA particles were well incorporated into the microspheres, while the number of HA particles increased with an increase in initial HA content in the collagen/HA solution (Figure 4A–C). In addition, some HA particles, indicated by the arrows, were observed with the collagen nanofibers (Figure 4D). This finding suggests that the HA particles can be effectively incorporated into the composite microspheres, while they are preferentially located at the surface of the microspheres, presumably due to the surface gelation process before entire self-assembly-induced gelation (cf. Figure 1A,C). An EDS analysis confirmed the chemical composition of the HA particles with strong peaks observed in association with Ca, P, and O (data not shown here). 

**Figure 5:**
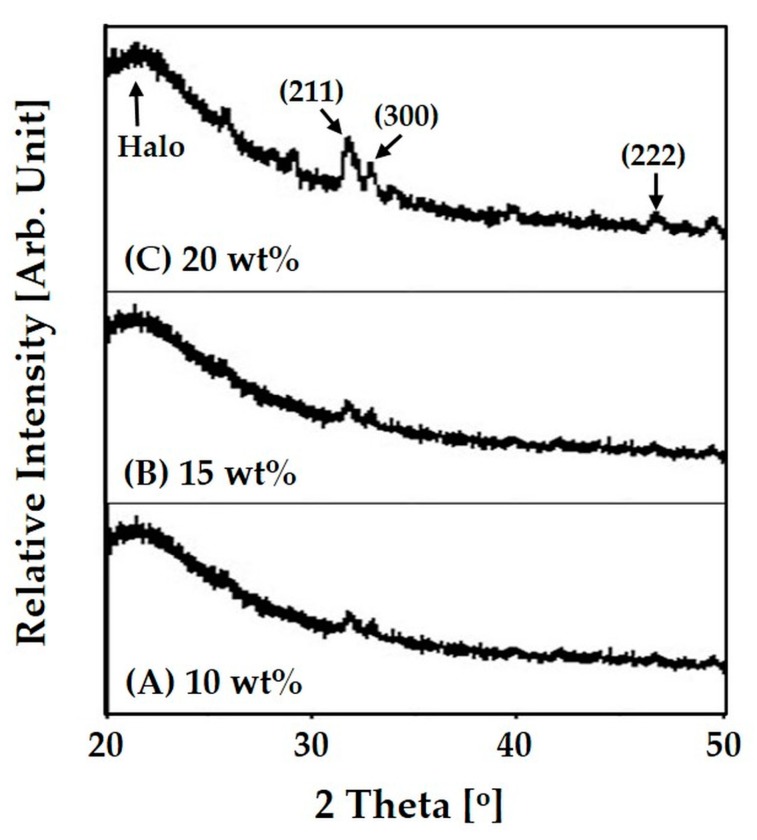
Representative X-ray diffraction (XRD) patterns of nanofibrous collagen/HA composite microsphere synthesized with different levels of HA: (**A**) 10 wt %; (**B**) 15 wt %; (**C**) 20 wt %.

The crystalline phases of the nanofibrous collagen/HA composite microspheres synthesized using different levels of HA content (10 wt %, 15 wt %, and 20 wt %) were characterized by XRD, as shown in Figure 5A–C. All the composite microspheres exhibited peaks corresponding to those of HA crystalline phases (JCPDS file No. 09-0432), while a halo peak at a low diffraction angle was observed due to the collagen phase. However, the relatively intensity of the peaks associated with (211), (300), and (222) planes of crystalline HA increased with a rise in initial HA content in the collagen/HA solution, indicating higher HA content incorporated into the composite microsphere.

Table 3 summarizes the final HA content in the composite microspheres, which was calculated by TGA analyses. The composite microspheres synthesized using collagen/HA solutions with high HA contents (15 wt % and 20 wt %) contained slightly less HA. However, it should be noted that most of the HA particles can be well incorporated into the collagen matrix during the emulsification process, which would provide significantly enhanced bioactivity and bone regeneration ability *in vivo*.

### 3.4. In Vitro Apatite-Forming Ability of Composite Microspheres

**Figure 6:**
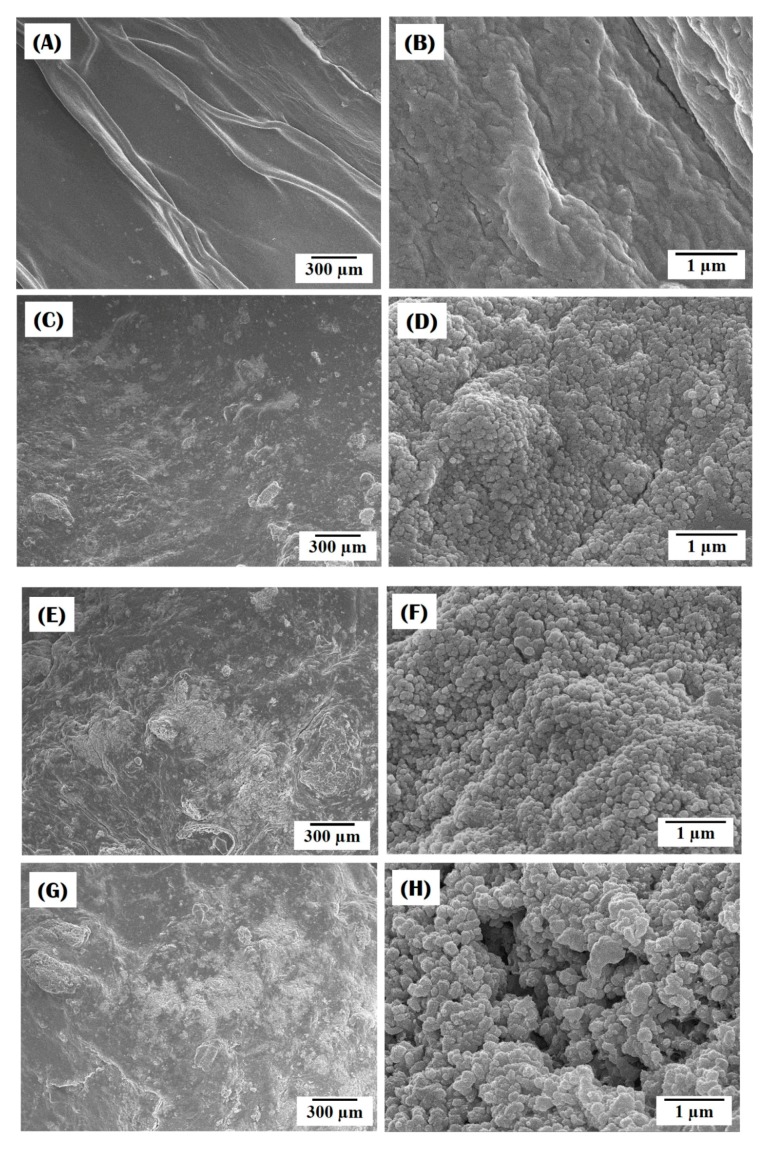
Representative FE-SEM images of nanofibrous collagen/HA composite microspheres synthesized with different levels of HA: (**A**,**B**) 0 wt %; (**C**,**D**) 10 wt %; (**E**,**F**) 15 wt %; (**G**,**H**) 20 wt %, after soaking in SBF solution for 3 days.

To evaluate the potential for nanofibrous collagen/HA composite microspheres to be used as bone scaffolds, their *in vitro* apatite-forming ability was examined using an SBF solution. Figure 6A–D display representative SEM images for composite microspheres synthesized using different levels of HA content (0 wt %, 10 wt %, 15 wt %, and 20 wt %) after 3 days of immersion in the SBF solution. The pure collagen microspheres maintained a relatively smooth surface even after soaking in the SBF for 3 days (Figure 6A) and revealed no sign of apatite crystal precipitation (Figure 6B). On the other hand, all the collagen/HA composite microspheres showed signs of the formation of apatite crystals on their surface, which appeared in bright contrast (Figure 6C,E,G). In addition, the area covered with apatite crystals increased with an increase in HA content. The bright area of the SEM image are comprised of a number of nano-sized apatite crystals (Figure 6D,F,H), indicating excellent *in vitro* apatite-forming ability. This finding suggests that the incorporation of HA particles into the collagen matrix can significantly enhance the *in vitro* apatite-forming ability of the collagen/HA composite microspheres and that higher HA content can lead to higher bioactivity. 

**Figure 7:**
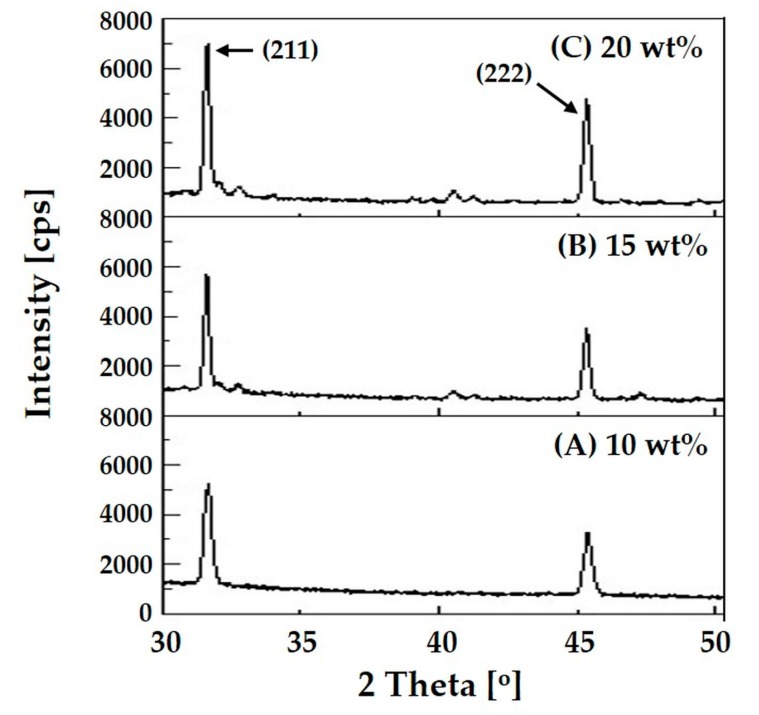
Representative XRD patterns of nanofibrous collagen/HA composite microsphere synthesized with different levels of HA: (**A**) 10 wt %; (**B**) 15 wt %; (**C**) 20 wt %, after 3 days of immersion on the SBF solution.

The crystalline phases of the apatite crystals precipitated on the composite microspheres were characterized by XRD, as shown in Figure 7A–C. After 3 days of immersion in the SBF solution, all the composite microspheres exhibited very strong, sharp peaks corresponding to those of HA crystalline phases. It should be noted that the intensities of these peaks were much higher than those observed for the as-synthesized microspheres (cf. Figure 5). This finding suggests that apatite crystals were vigorously precipitated on the surface of the composite microspheres after immersion in the SBF solution. The intensity of the peaks corresponding to (211) and (222) planes of crystalline HA increased with an increase rise in HA content. This finding suggests that higher HA content in the composite microspheres can provide higher apatite-forming ability *in vitro*.

### 3.5. Utility of Self-Assembly-Induced Gelation

**Figure 8:**
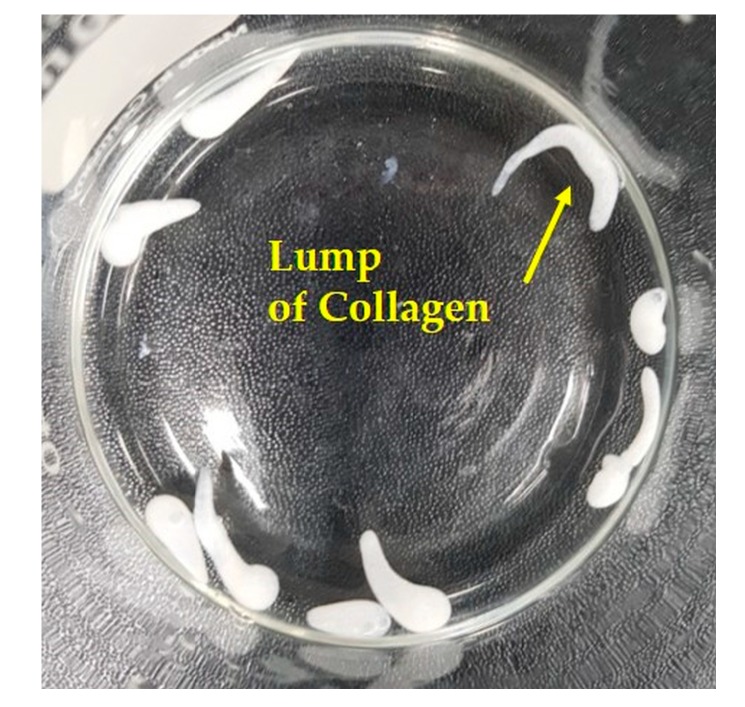
Optical image showing the lumps of collagen synthesized using conventional emulsion technique.

The self-assembly-induced gelation technique, introduced for the first time in this study, has several unique features, which are difficult to obtain using conventional techniques for the synthesis of microspheres. First, this technique can have great ability to achieve very consistent size uniformity, particularly through the controlled formation of a collagen droplet at the nozzle tip in air, followed by the surface gelation of the droplet in a coagulation bath before extensive pH-dependent precipitation throughout the microspheres (cf. Figure 1A,B). For example, when a collagen solution was directly added to a coagulation bath under vigorous magnetic stirring, which is similar to the conventional emulsion technique, a lump of the collagen solution with an irregular shape was formed due to fast gelation before making spherical droplets (Figure 8). Thus, the surface gelation of collagen droplets in a coagulation bath before extensive pH-dependent precipitation is a crucial step to achieve a well-defined spherical shape. 

**Figure 9:**
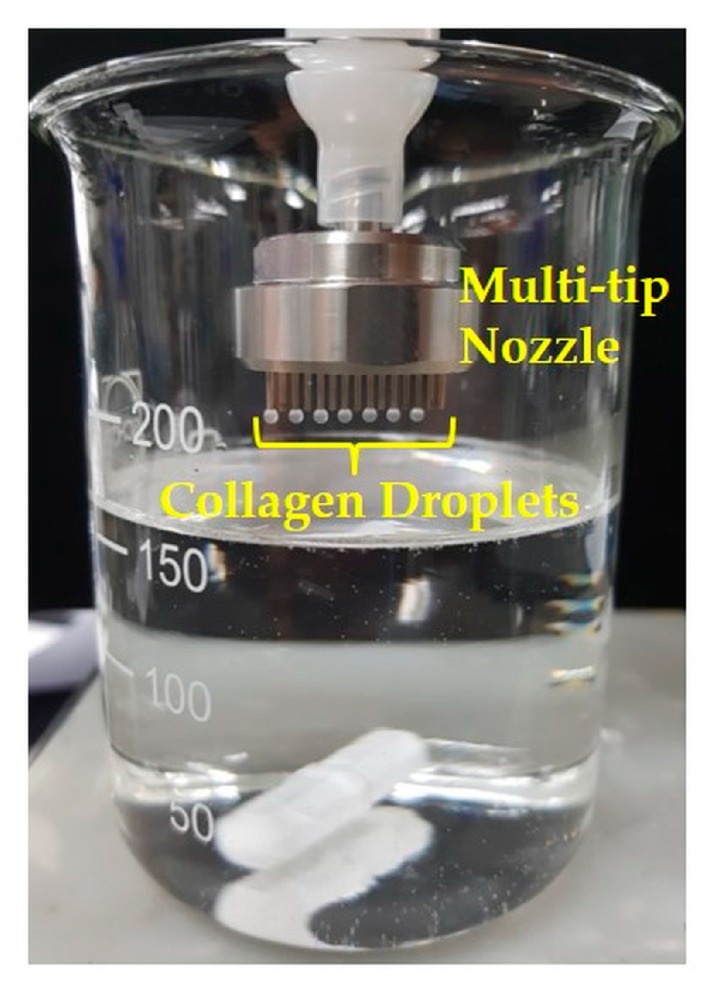
Optical image of collagen droplets formed at the tips of a multi-tip nozzle.

Second, a highly nanofibrous structure, resembling that of natural ECM, can be created through the self-assembly of collagen aggregates into fibrils via pH-dependent precipitation in the coagulation bath. It should be also noted that a multi-tip nozzle can be used to increase the yield, as shown in Figure 9. 

**Figure 10:**
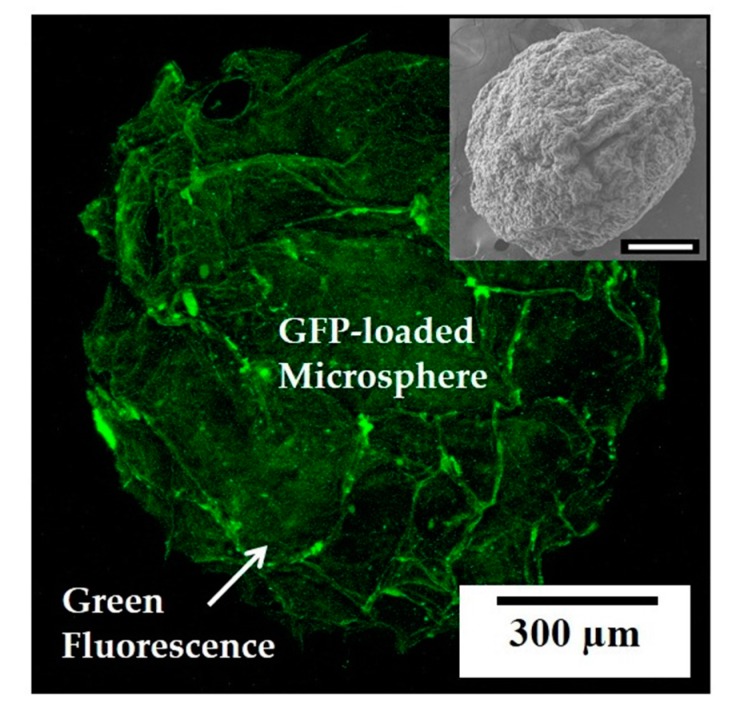
Representative CLSM image of the green fluorescent protein (GFP) oaded collagen microsphere. The inset shows the SEM image of the GFP-loaded collagen microsphere (scale = 300 μm).

Third, the present approach is very useful to synthesize bioactive molecule-loaded collagen microspheres simply using collagen solutions containing bioactive molecules. To demonstrate this, green fluorescent protein (GFP) loaded microspheres were synthesized using a collagen solution containing the GFP (inset in Figure 10). CLSM observation revealed that a uniform distribution of strong green fluorescence was detected throughout the microsphere (Figure 10). This finding suggest that the GFP can be effectively, uniformly loaded into the nanofibrous collagen microsphere without noticeable loss during the entire process. It should be noted that a variety of biomolecules, such as growth factors and drugs, would be also incorporated into nanofibrous collagen microspheres using the present self-assembly-induced gelation approach. 

**Figure 11:**
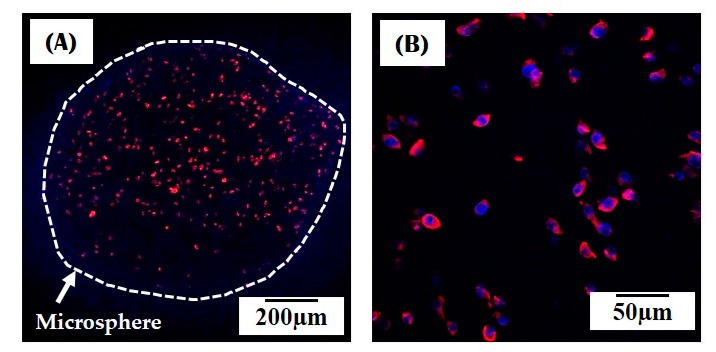
Representative confocal laser scanning microscopy (CLSM) images of the cell-loaded collagen microsphere: (**A**) Low magnification; (**B**) High magnifications.

In addition, the present approach would allow for the direct incorporation of living cells into collagen microspheres, although conditions for gelation and cross-liking processes would be required to be modified for high cell survival. The MC3T3-E1 cells were uniformly distributed throughout the collagen microspheres (inset in Figure 11). In addition, the cells were metabolically active and viable, where the red and blue colors represent the actin and nucleus, respectively (Figure 11). However, it should be noted that a further *in vitro* study should be carried out to evaluate the behavior of the cells incorporated into the microspheres, including cell proliferation, and differentiation, which would clarify the utility of the cell-loaded microspheres for bone tissue regeneration.

It should be noted that the newly developed self-assembly-induced gelation would be used with a variety of polymer solutions and biomolecules solutions that can be gelled in a coagulation bath. In particular, microspheres made of biomolecules would be expected to be used as templates to induce the precipitation of apatite crystals, thus finding very useful applications in bone tissue engineering [33].

## 4. Conclusions

Nanofibrous collagen/HA composite microspheres with different levels of HA content (0 wt %, 10 wt %, 15 wt %, and 20 wt %) were successfully synthesized using self-assembly-induced gelation—newly developed in this study. All the composite microspheres had similar diameters with a narrow size distribution, ranging between 798 μm ± 42 μm and 809 μm ± 34 μm. In addition, regardless of the HA content, collagen nanofibers—several tens of nanometers in size—were uniformly created throughout the composite microspheres. The HA particles were also successfully incorporated into the composite microspheres, which significantly enhanced *in vitro* apatite-forming ability. A number of apatite crystals vigorously precipitated on the surface of the composite microspheres after 3 days of immersion in an SBF solution. It was also found that higher HA content led to higher *in vitro* bioactivity. These findings suggest that self-assembly-induced gelation could be used for a variety of polymer solutions and be applied in a number of different fields.

## Figures and Tables

**Table 1:** Ion concentrations of the stimulated body fluid (SBF) used for the *in vitro* apatite-forming ability test.

Ion	Na^+^	K^+^	Mg^2+^	Ca^2+^	Cl^−^	HCO_3_^−^	HPO_4_^2−^	SO_4_^2−^
Concentrations (mM)	142.0	5.0	1.5	2.5	148.8	4.2	1.0	0

**Table 2:** Diameters of the nanofibrous collagen/HA composite microspheres synthesized using different levels of HA content (0 wt %, 10 wt %, 15 wt %, and 20 wt %) in the collagen/HA solutions.

HA Content [wt %]	0	10	15	20
Diameter [µm]	802 ± 40	798 ± 42	806 ± 38	809 ± 34

**Table 3:** Final contents of the HA phase in nanofibrous collagen/HA composite microspheres synthesized using different levels of initial HA content (0 wt %, 10 wt %, 15 wt %, and 20 wt %) in the collagen/HA solutions.

**Initial HA Content [wt %]**	10	15	20
**Final HA Content [wt %]**	10.4	13.3	17.6
